# Stochastic Resonance Crossovers in Complex Networks

**DOI:** 10.1371/journal.pone.0051170

**Published:** 2012-12-14

**Authors:** Giovanni Pinamonti, J. Marro, Joaquín J. Torres

**Affiliations:** Institute “Carlos I” for Theoretical and Computational Physics, and Department of Electromagnetism and Matter Physics, University of Granada, Granada, Spain; Humboldt University, Germany

## Abstract

Here we numerically study the emergence of stochastic resonance as a mild phenomenon and how this transforms into an amazing enhancement of the signal-to-noise ratio at several levels of a disturbing ambient noise. The setting is a cooperative, interacting complex system modelled as an Ising-Hopfield network in which the intensity of mutual interactions or “synapses” varies with time in such a way that it accounts for, e.g., a kind of fatigue reported to occur in the cortex. This induces *nonequilibrium* phase transitions whose rising comes associated to various mechanisms producing two types of resonance. The model thus clarifies the details of the signal transmission and the causes of correlation among noise and signal. We also describe short-time persistent memory states, and conclude on the limited relevance of the network wiring topology. Our results, in qualitative agreement with the observation of excellent transmission of weak signals in the brain when competing with both intrinsic and external noise, are expected to be of wide validity and may have technological application. We also present here a first contact between the model behavior and psychotechnical data.

## Introduction

Ambient fluctuations that are treated as annoying and often ignored play in fact a fundamental role in nature. For example, they may transmit information notwithstanding their deceptive lack of structure (see, e.g., [1], [2]), help setting up order at the macroscopic, mesoscopic and even nanoscopic levels despite their apparent order-disturbing effect [3], and optimize propagation by turning the medium into an excitable one [4], [5] and inducing coherence among environmental noise and the periodic part of the signal, which helps weak inputs to go through without damping. This is named *stochastic resonance* (SR) which, believed to occur in many different instances [6]–[15], and known to be technologically relevant, e.g., in designing filters and sensory devices and in extracting details about waves-traversed geological media [16], [17], is now established as a genuine and common, perhaps universal phenomenon [18]–[20].

Deciphering the detailed microscopic mechanisms bringing a constructive role of diverse fluctuations in such a varied range of circumstances is puzzling. This goal became even more difficult after the discovery of *stochastic multi-resonance* (SMR) in human perception [21] in accordance with predictions in assorted contexts, which demands searching for further causes [22]–[27]. The hallmark of SR is a rise of the power spectral density or the input-output correlation with increasing strength of a noise which is competing with the main input signal. The noise tends again to dominate, so that the signal transmission may be impeded in practice, past a peak as the noise level is further increased. One speaks of SMR when several peaks of this kind show up for different levels of noise.

In this paper, we report on a numerical study of SR and SMR in the Ising system on a network in which each node is linked to each other. Such a full wiring is not realistic but this feature is in practice swept away here by assuming inhomogeneous connectivity. That is, the interactions or connections are weighted and time varying following a pattern which has been observed, for instance, in the central nervous system [28]–[31]. This transforms in practice the original regular net into an effective complex network whose links happen to play an essential role, as described in detail, for example, in [31] and references therein. The ambient noise is modelled in our case by the standard thermal bath, and an external deterministic, time-periodic signal is added to the current arriving each unit. Using this simple setting, in which one may think of units and connections as oversimplified neurons and synapses, respectively, we describe a crossover from SR to SMR by changing the dynamic properties of synapses. Important features of SMR phenomena are then tuned by simply modifying model parameters that have a well-defined physical meaning. Our study thus deepens on the microscopic basis and therefore on the detailed nature of SMR as it may occur in an ample family of complex, cooperative or interacting systems, and we relate SMR to nonequilibrium phase transitions that are known to bear relevance to the understanding of some brain functions [32], [33].

## Methods

Let 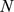 binary neurons, namely, 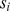 = 0 or 1, 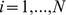, each linked to the rest by synapses 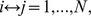 whose intensities or weights are given by the *covariance rule* [34]:
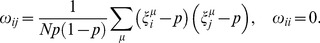
(1)This, which modifies the familiar Hebbian prescription to avoid saturation of weights, as if there were a threshold, involves 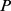 patterns, namely, 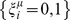 with 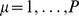, that are assumed to have been previously “learned” by the system. The parameter 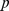 in (1) measures the excess of 1's over 0's or symmetry in the mean net activity of the set of patterns, namely, 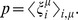 In practice, for simplicity and also to avoid specificities concerning this model feature, we deal here with random patterns in the sense that each 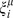 is given either 0 or 1 at random with the only restriction that 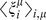 equals the given value of 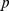.

Evolution with time is by parallel, cellular automata dynamics, namely, by stochastic changes at each time of the whole set 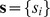 according to the probabilities:
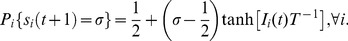
(2)Here, 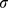 equals either 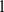 or 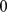, 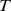 is the temperature of the underlaying bath, and
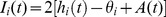
(3)stands for the total input on each neuron. The last term in this equation is an external signal that we shall first assume to be 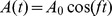 (see, however, the section “Changing the signal” below) where the amplitude 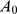 will in practice be small compared to the total input, and 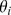 are thresholds for firing, which we take here equal to half the sum of the weights of all the synapsis connecting 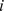 to the other neurons, 
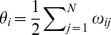
. The first term in the rhs of Eq. (3) is the net current from others on neuron 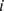, which is given by
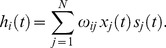
(4)Therefore, we modulate the synaptic weights with the variable 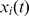 that we shall assume to change with time according to the map [28]:
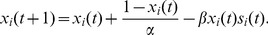
(5)This ansatz could be replaced by direct assumptions on the net links that have an easy interpretation on physical grounds, see e.g. [31], without affecting our main results here. Nevertheless, the choice (5) is simpler and has been previously tested in neuroscience studies [35]. It amounts to assume a sawtooth–shaped time change, with 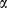 and 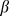 measuring the teeth width and depth, respectively, describing a competition of effects associated to synapses “fatigue”. That is, the link of intensity 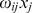 is debilitated as 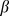 is increased, while decreasing 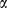 makes 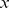 to recover its maximum value more rapidly. The link weight effectively remains constant in practice if such a recovery becomes very fast, so that one sometimes speaks of “
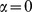
” as the limit of static synapses which characterizes the standard Ising and Hopfield cases [36], [37]. The origin of (5) are differential equations trying to account for the fact that electrical stimulation due to local and even spatially extended activity may induce short-term plasticity leading to *depression* and sometimes also *facilitation* of synaptic transmission [35], [38].

The relevant order in this system may be described by monitoring the firing rate, i.e., 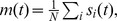 which is in fact sometimes recorded in laboratory experiments. Though hardly experimentally accessible, also interesting to illustrate in detail the system behavior is the overlap of the actual state with each pattern 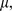 defined as
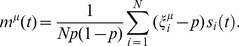
(6)Furthermore, we are interested in measuring the intensity of the input-output correlation, so that we shall compute the function
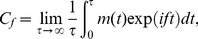
(7)i.e., the Fourier coefficient at frequency 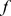 of the output firing rate. The relevant correlation, to be denoted 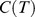 in the following, is signal dependent, e.g., we define it in the cosinus case as the value of 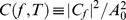 computed at the frequency of the input signal.

The phase diagram of the above model with 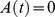 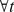 was examined before [28], [29], [31], [32], [39]. The most detailed study so far concerns the case in which 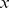 in (4) is interpreted as a stochastic variable with distribution inspired in (5) [31]. A main result in this case, which does not differ essentially from the present one, is its relevance to better understanding cooperative phenomena in several fields. In particular, tuning properly parameter values, the model exhibits familiar equilibrium phases, namely, a disordered high-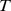 phase —corresponding to the *paramagnetic* phase in condensed matter— in which (the stationary values of) all the overlaps are practically zero, a low-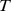 phase with conventional order —corresponding to *ferromagnetism*— in which the global activity converges with time towards one of the attractors 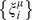, so that it is often taken as a model example of associative memory, and a —say, *spin-glass*— phase in which convergence is towards a mixture of stored patterns. In addition, the system may be tuned to exhibit nonequilibrium phases [36]. Namely, (*i*) one in which there is a rapid and rather irregular roaming among the attractors —thus closely mimicking, for example, long-time structural changes and oscillations that have been associated with reaction–diffusion phenomena in physics and chemistry, as well as efficient, say, *states of attention* that are of interest in neuroscience—, (*ii*) one which is mainly characterized by oscillations between one of the stored patterns and its negative or corresponding *antipattern*, and (*iii*) one with quite irregular, apparently chaotic roaming randomly interrupted by pattern–antipattern oscillations [31]. The case (5) induces similar though relatively simpler behavior, e.g., the most involved behavior (*iii*) does not seem to fully develop in this case.

## Results

### From single to multiple resonance

We report here on Monte Carlo simulations of the above model. Exploratory runs showed no essential influence of 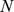 nor 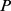 in the main behavior of interest, so that we shall report first on the *sufficiently large*, typical case 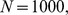 and will focus on 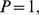 i.e., the only dynamic attractors are a given pattern and its antipattern. Varying 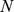 and 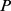 is also interesting, however, and we shall latter be concerned with this. The stored pattern will initially correspond to 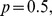 which means same number of firing and silent neurons on the average, but changing 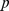 will be shown later on to modify importantly the system behavior. Time series for performing averages consisted of 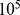 Monte Carlo steps.

**Figure 1:**
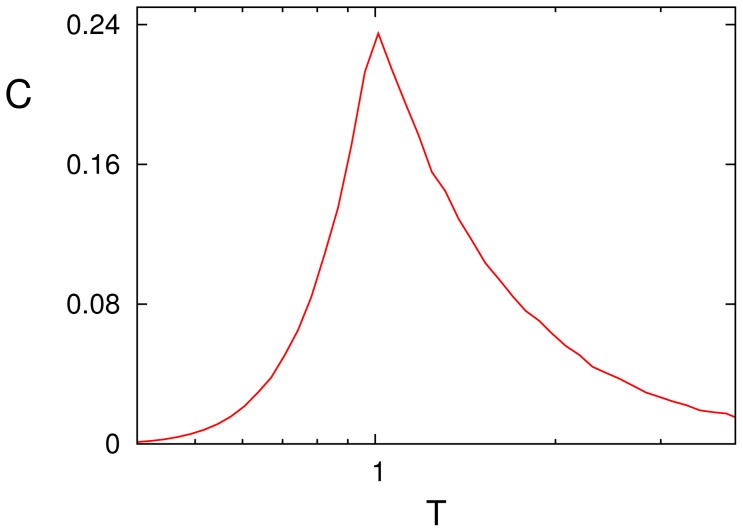
The signal–to–noise function 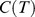
 **depicts in this semilogarithmic plot a shallow resonance for static synapses at the critical temperature.** (Here, 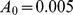, 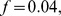 and 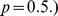

**Figure 2:**
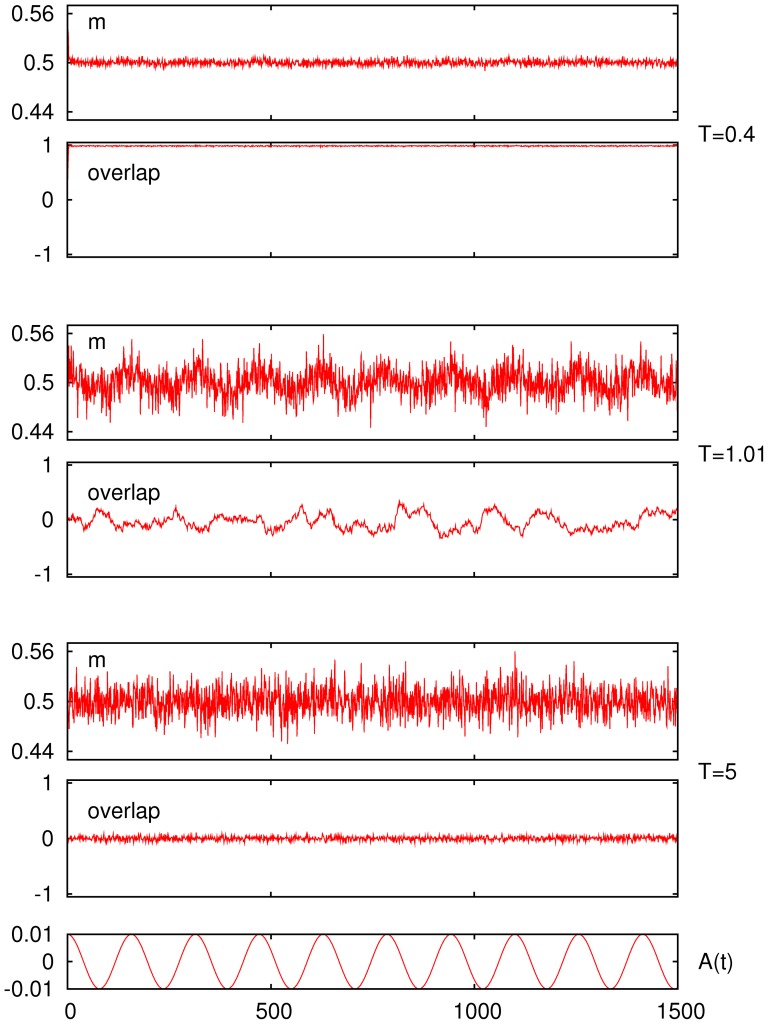
Three sets — at different noise level or *temperature* 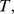
 **as indicated — each with two time series for, respectively, the firing rate (top of each set) and the overlap (bottom of each set) showing a tendency towards coherence at** 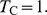 The common external signal 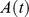 and time scale are shown at the bottom below the sets. (Same case as in Fig. 1, except that *A* 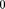 = 0.01.)

In the Hopfield limit of static synapses, 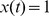 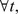 the system exhibits a rather weak resonance. As shown in Fig. 1, a well-defined though shallow peak in the input-output correlation occurs around 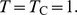 This is the bath temperature separating the ferromagnetic phase, for 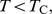 from the disordered phase, for 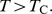 The mechanism behind this behavior is illustrated in Fig. 2. This exhibits typical time series corresponding to the two relevant equilibrium phases. Namely, one is characterized by non-zero overlap —in fact, this is close to its maximum in our example shown as the second graph of the top set for 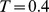
— and the other by zero overlap —i.e., small-amplitude fluctuations around zero as in the bottom set. This figure also exhibits a near-critical condition (middle set) in which the overlap shows larger-amplitude fluctuations. It is remarkable that only in the latter case with 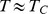 is the firing rate clearly coupled to the cosinus within 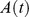; the overlap also happens to be somewhat coupled here to the signal but this is not obvious to the naked eye in Fig. 2. The familiar critical bistability resulting from a competition between *thermal* fluctuations and —static though non-homogeneous— node interactions is in this case the mechanism [18], [19] that allows the weak signal to prevail despite the noise.

More involved behavior shows up when synapses are dynamic, namely, 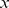 in (4) varies with time as stated in (5). As a matter of fact, one may then expect changes in the transmission of signals, given the very different development of order which occurs depending on the parameter values in this case, as we described at the end of the previous section.

**Figure 3:**
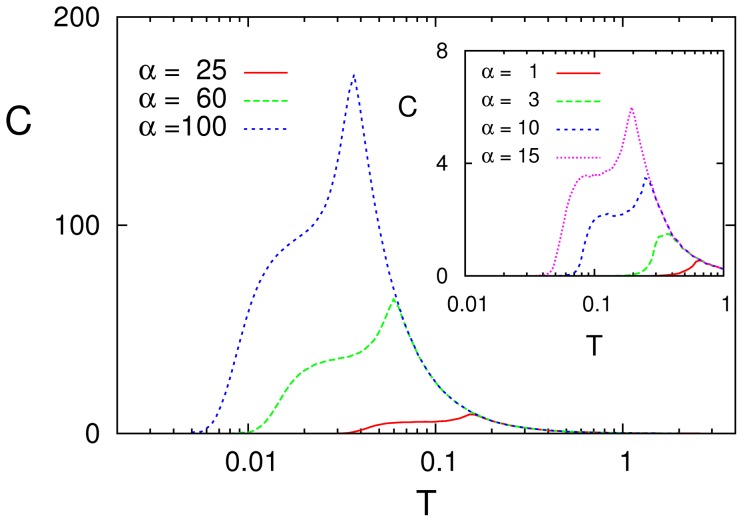
Different resonance curves 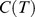
 **as one modifies the value of** 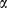 **in (5), as indicated, for** 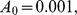 **** 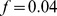 **and** 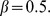

Fig. 3 illustrates the case as one modifies the depression parameter 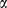 in (5). The SR maximum is still clearly depicted for any 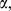 but it corresponds now to the transition between the equilibrium disordered phase and the nonequilibrium one characterized by (possibly irregular) oscillations of the global activity —that is, the phase identified (*ii*) above. Furthermore, two other main differences arise. One is that the peak location moves as 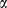 increases towards lower temperature, in agreement with a reported scaling of the critical temperature with synaptic depression [28]. Furthermore, there is a factor of near 10^3^ in the vertical scale here as compared to the one in Fig. 1, namely, the resonance effect is now much stronger, though the signal for this figure is even weaker than in the simulation before for static synapses.

Actually more intriguing is some indication of SMR for dynamic synapses, i.e., 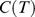 tends to form and sometimes develops a plateau at low temperature which seems to announce a second resonance peak having a different origine that will finally show up for 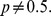 The tendency is not fully materialized here, however, due to our restriction so far to strictly symmetric patterns (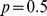), which induces some symmetry of the connection intensities, as we discuss next.

### Effects of asymmetry

The fact that the incipient correlation plateaus in Fig. 3 are associated to the mechanisms inducing transitions between the equilibrium-memory and nonequilibrium-oscillatory phases is confirmed by analysis of the corresponding time series (not shown). That is, one observes that the overlap then describes rapid oscillations between the stored pattern and its antipattern that are definitely correlated with the signal waving. Closer inspection does not evidence any such correlations in the firing rate series, however. Consequently, the function 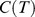 —which derives from 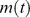
— shows no definite peak. This apparent inconsistency is because, in as long as one considers 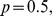 the firing rate, unlike the overlap, fluctuates with only small amplitude, around 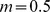 in practice. It follows that analyzing 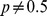 is needed now, specially after one notices that the asymmetric case is in fact the only bearing interest for hypothetical realizations of this resonance phenomenology in the laboratory.

**Figure 4:**
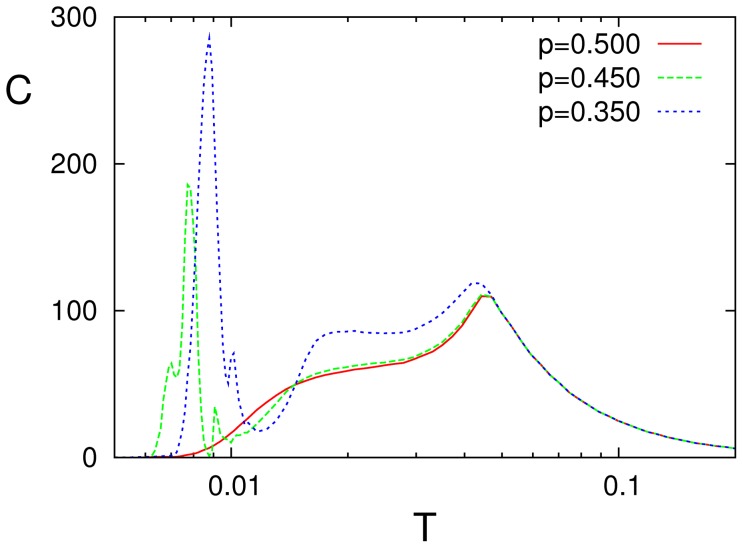
Resonance curves when one introduces an essential asymmetry by varying the mean neuron activity in the stored pattern, 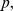
 **as indicated.** (Here, 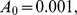 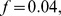 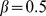 and 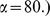

**Figure 5:**
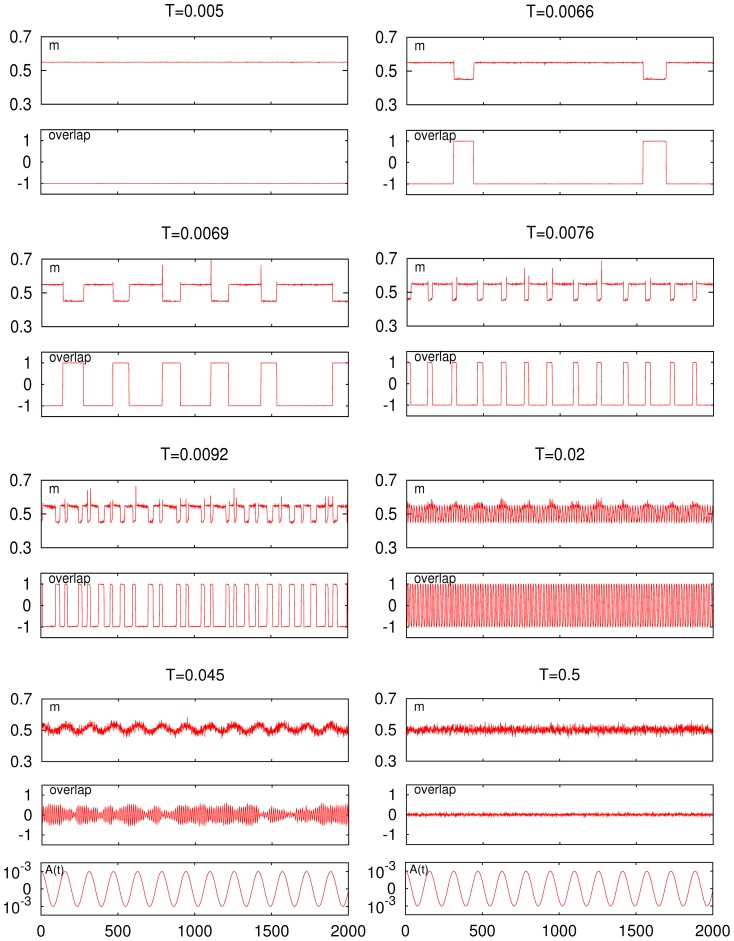
Time series for the firing rate (top graph of each set) and for the overlap (bottom graph of each set) at different temperature, as indicated, in the asymmetric case 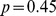
 **.** (Other parameters as in Fig. 4.) The second set from top in the right column corresponds to the low-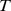 peak; the bottom set in the left column corresponds to the high-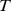 peak. The common external signal 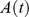 and time scale are shown at the bottom below the sets.

Figs. 4 and 5 illustrate the change of behavior as the mean neuron activity in the pattern, 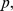 is modified. The first one shows that any asymmetry in the number of firing and silent neurons induces SMR, namely, a sharp peak (together with some “harmonics”) at very low 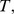 near the transition between memory and oscillatory phases, and a cleaner and somewhat less pronounced peak at higher 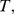 near the transition between oscillatory and disordered phases. Interesting enough, the resonance is enhanced with increasing asymmetry. We also notice that, as expected, the underlying pattern-antipattern symmetry induces the same behavior for 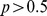 than for 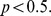


Fig. 5 clearly depicts the nature of the low-temperature resonance peak and how this is associated with asymmetry. That is, the oscillations of the firing rate are essentially different for the two cases of correlated behavior. One observes at 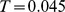 a behavior that resembles the one for the middle set in Fig. 2. This is a critical condition, corresponding to a second–order phase transition, in which the resonance is essentially induced by noise and long–ranged correlations. There are oscillations of both 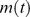 and 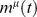 that are definitely correlated with those of 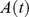 —which results in the high-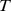 resonance peak— but occurring between states that, due to the underlaying noise, are not strongly correlated with the information content, as one should have expected given that jumping is now practically among the store pattern and a disordered phase. Perhaps the most striking observation here is that 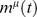 subtly correlates with the signal, namely, it occurs as a modulation in the amplitude of the pattern–antipattern oscillations (see middle panel of the bottom left set in Fig. 5). Also interesting is that, in spite of the noise in this case, the weak signal is able to correlate with the neurons activity therefore affecting the processing of information at very short time scales, as discussed further in the next section.

The relevant mechanism happens to be qualitatively different near the low-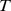 resonance peak, e.g. 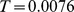 in Fig. 5. Both the firing rate and the overlap now show abrupt oscillations with precisely the same frequency and strongly correlated with 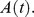 In particular, the low (high) firing metastable states corresponding to high (low) overlap —i.e., transitions between the two only possible levels of neural activity in the (normal) case of asymmetric patterns— are synchronized to the maxima (minima) of the cosinus signal. As in a first–order phase transition, and unlike the high-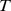 case, such a strong correlation tends to diminish sharply as 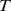 is either increased or decreased even slightly, Fig. 5 reveals. Furthermore, none of the time series, 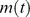 and 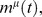 display superimposed fluctuations, confirming that the noise, even though necessary, is not here the relevant cause. The control is now in the weak signal, and the global activity changes correlated with the information content during a relatively long time, namely, one at least of order of the signal period.

**Figure 6:**
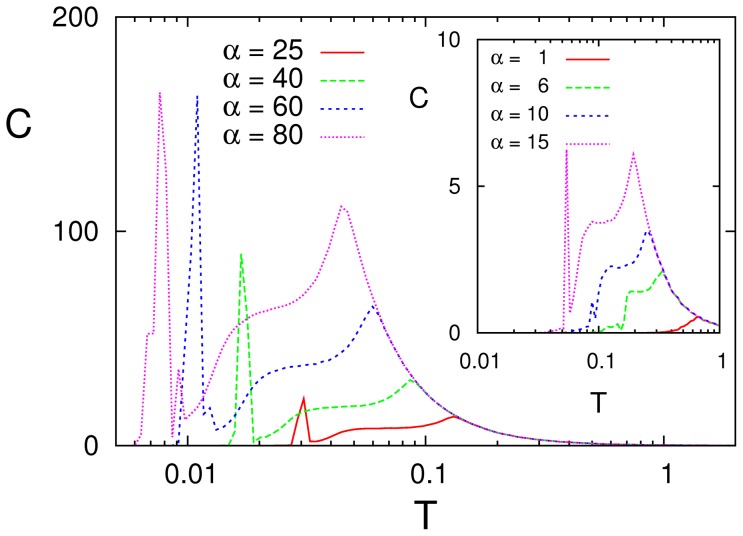
Resonance curves for varying 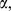
 **as indicated, when** 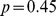 **and** 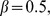 **for a sinusoidal signal with** 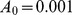 **and** 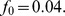

Fig. 6 illustrates the situation for 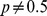 as one changes 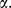 On one hand, the behavior happens to be similar to the one for SR as observed above in the symmetric case (cf. Fig. 3), namely, increasing (decreasing) 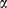 shifts the peaks to lower (higher) 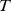 and, at the same time, the high of the peak increases (decreases). On the other hand, the two peaks tend to merge into a single one as 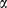 is decreased, and the low-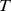 peak does not really show up in practice for any 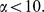 A main conclusion is therefore that SMR requires both asymmetry of the patterns concerning 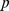 in (1), which is in fact a general property of nature, and large enough values of the parameter 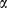 characterizing the synaptic changes in (5), i.e., a complex functionality of connections —even though the actual wiring may be a simple, fully-connected one.

### Changing the signal

**Figure 7:**
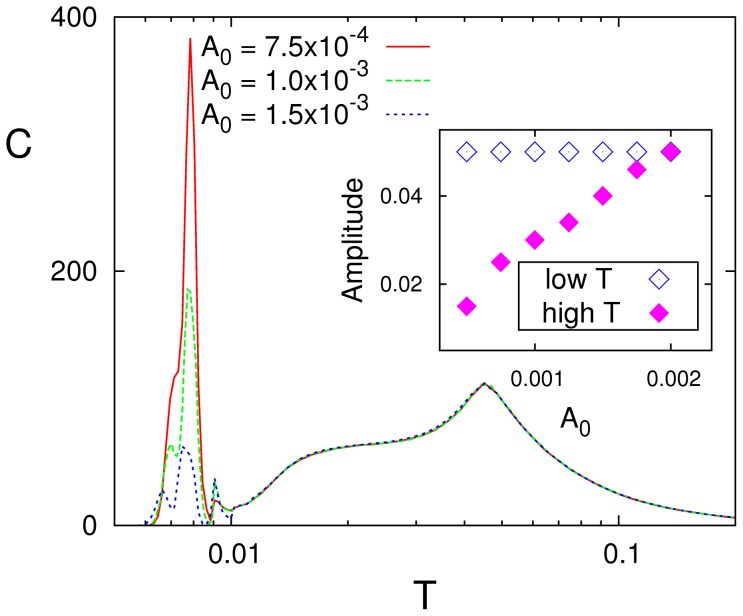
Effect of varying the amplitude 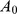
 **for** 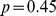. The inset shows the dependence on 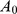 of the amplitude of the oscillations of 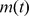 for each of the two peaks.

The above suggests that the details of the input signal may also have an effect on resonance. Indeed, Fig. 7 reveals a substantial influence of the amplitude 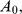 and confirms the different nature of the two peaks. While the high-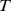 peak remains constant, the low-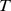 peak strongly increases with 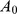 for 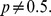 This is due to the normalization of 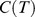 with respect to 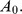 That is, since the oscillations that correspond to the first peak are fixed in amplitude (the system is switching between pattern and antipattern), the normalization factor leads to the inverse dependence between the peak height and the signal amplitude. This is not the case for the second resonance peak because the amplitude of the oscillations in the firing rate also increases with 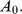 This peak of 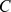 thus remains constant, maintaining its shape and height independently of the value for 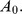 Such differences are a consequence of what we observed above in relation with Fig. 5. That is, the behavior around 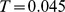 is determined more by the signal —and, therefore, by 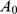
— than by the well to be overcome at the transition point, while the well depth dominates over the signal influence around the (first–order) transition in 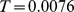.

We also checked the robustness of behavior in relation to the nature of the signal. Let us consider, which is a familiar case, a non-homogeneous Poissonian spike train with an instantaneous firing rate modulated by a slow sinusoidal function. That is, instead of a cosinus, we shall now use in Eq. (3) the signal 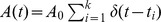 were the occurrence times 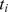 are generated from a non-homogeneous Poisson process of mean 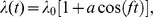 i.e., varying with time. This is believed to be more realistic than a sinus or a cosinus, at least for neural systems, e.g., this is sometimes assumed to represent the spike activity of a neuron in sensory areas processing structured external signals from senses. This choice is also a more general function, which eliminates specific features of the cosinus and includes both stochasticity (inherent here to the Poisson process) and some quasi–periodic structure codifying relevant information, which is important for the involved phenomena.

**Figure 8:**
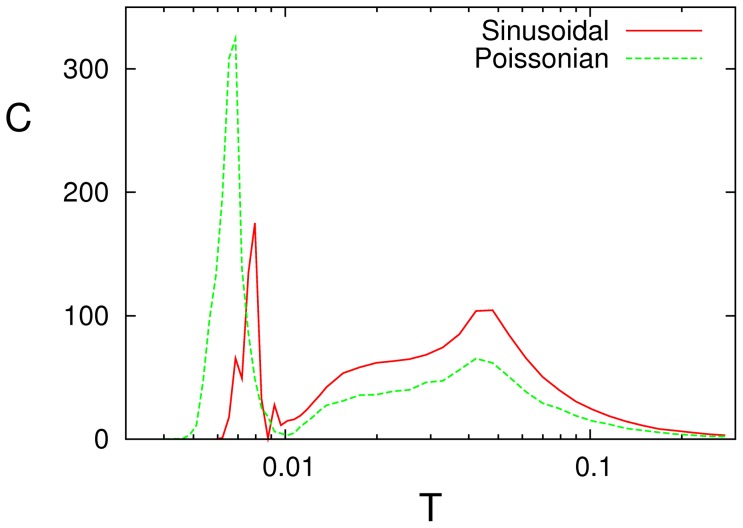
Resonance curves for a sinusoidal signal and for a non-homogeneous Poissonian input train (in this case, 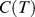
 **stands for** 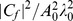 **at the modulation frequency** 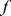 **of the non-homogeneous Poissonian process rate).** Here, 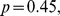 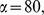 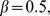 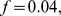 and 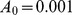 for the sinus and 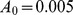, 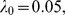 and 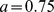 for the Poissonian signal.

**Figure 9:**
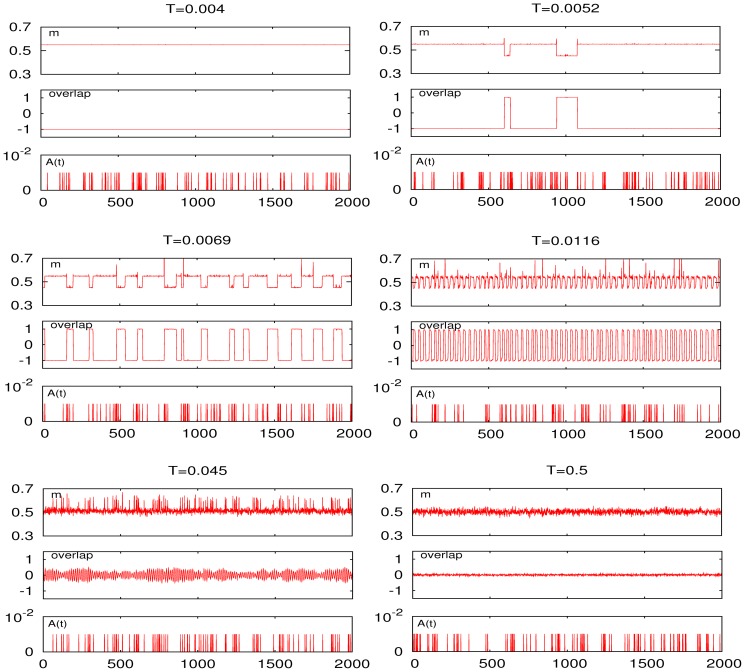
Time series for different values of 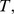
 **as indicated, corresponding to the SMR curve in** **Fig. 8** **for the Poissonian input train (shown below each set with the time scale).** The resonances occur in this case around 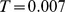 (second set in the left collumn) and 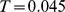 (third set in the left column).

A first observation is that, as Fig. 8 illustrates, no essential qualitative changes occur using one or the other signal in a typical case of SMR. On the other hand, inspection of time series as those in Fig. 9 shows again indications of the different nature of the two peaks. At low 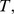 e.g., 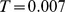 in this figure, the firing rate switches from low to high mean activity each time a train or burst of inputs arrives. Once the stimulus ends or the arriving signals become sparse, the system stays at the metastable state of high activity —as it occurs in Fig. 5 for the cosinus maxima— until synapses depress, due to such staying at high activity, and the metastable state destabilizes. It seems sensible to link this behavior with that in a hypothetical working memory context in which the activity persists for some time after the stimulus has ceased. As a matter of fact, a sort of short–term synaptic plasticity which reminds one of this situation has already been proposed 40,41. On the contrary, the system processes without slothfulness at high 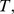 e.g., 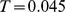 in Fig. 9. That is, a single spike input induces switching from low to high activity, and the high activity state persists but only during the duration of the stimulus, so that any temporal structure encoded in the signal is precisely processed at the high-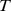 resonance.

## Discussion

We here studied the origin of stochastic resonance as it occurs in a biologically-motivated Ising-Hopfield model system with thresholded neurons and dynamic synapses. This results in an interacting complex network, namely, one in which the intensity of connections is inhomogeneously distributed and varies with time, which essentially influences functionality. For a wide range of parameter values, the system shows intense resonance for different levels of noise. More specifically, as the noise is increased in case 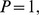 i.e., when the system *stores* a single pattern, the network activity passes from a resting state with some activity around this pattern to a phase in which this situation destabilizes and the global activity oscillates between the metastable states corresponding to the pattern and its antipattern configurations. When the noise increases even more, the pattern–antipattern oscillations wash out and a disordered phase emerges. Interesting enough, SMR happens to require in this setting some synaptic depression, so that the relevant phases occur —and the stored pattern to be asymmetric as it is always the case in practice. Two resonance peaks —namely, sudden increase of the efficiency in transmitting a weak signal through two different levels of the environmental noise— are then exhibited that are associated with the transitions points between the phases.

The nature of the peaks importantly differs from each other. The low noise one is mainly due to the coupling between the frequency of the pattern–antipattern oscillations —associated to the occurrence of nonequilibrium phases— and the waving of the input signal. The high noise peak, however, ensues when a modulation of the amplitude of these oscillations (and not the pattern–antipattern oscillations themselves) correlates with the signal. This relevant modulation clearly manifests itself as a noisy slow oscillation in the firing rate, as illustrated by the inset of Fig. 7 showing how the amplitude of the firing rate oscillations increases with the amplitude of the signal.

The peaks not only differ in their birth mechanism but also in the way the signal is processed. This is made evident when an inhomogeneous Poissonian spike train of small amplitude is used as input signal. Around the low-noise peak, the system activity rather tends to follow the signal every time a burst of spikes arrives, and it remains excited for a time, which is short but larger than the stimulus duration, until the synaptic fatigue mechanism destabilizes such metastability. This is precisely the basic microscopic origin of peculiar properties reported to occur in nature such as undamped propagation in excitable media [4], [32], [33], and it may also be interpreted as a sort of short–term memory mechanism able to maintain information for, say, a few seconds as in the so–called sensory and working memories. The situation essentially changes around the high-noise peak, where the system detects each single input spike, that is, the finest time structure of the underlying signal.

**Figure 10:**
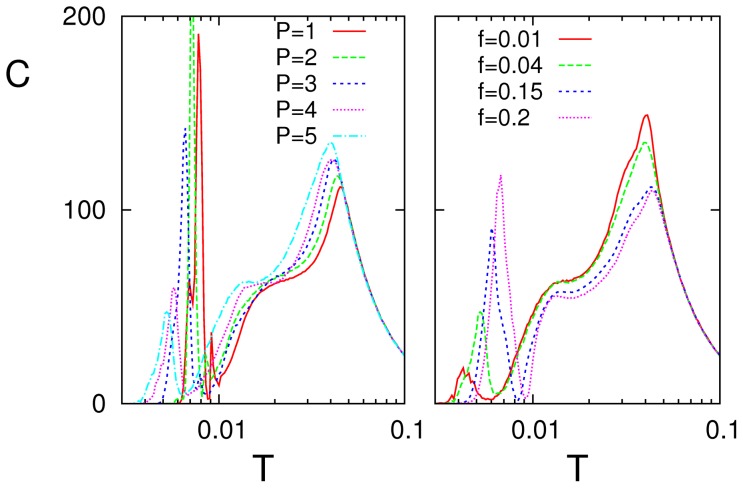
Left: Resonance curves for 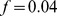
 **as the number** 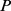 **of stored patterns is varied, suggesting that the low-** 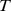 **resonance tends to disappear with increasing** 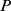. Right: Resonance curves for 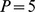 as one varies the signal frequency 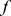. This shows the contrary effect, i.e., the low-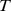 resonance intensity increases with 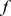. (Here, 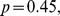 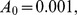 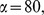 and 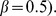

We also checked how SMR is affected by varying the number 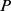 of stored patterns. This is interesting for completeness but also because the global activity becomes for 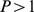 even more complex. That is, the system then tends to keep visiting all the stored patterns and their antipatterns, and it may do this by following quite irregular, even chaotic paths [31]. As Fig. 10 shows, increasing 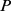 for a fixed frequency 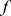 of the input signal (left), the high–noise resonance slightly increases and moves a little bit towards lower 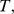 and the low-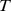 peak markedly decreases while moving to lower 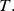 This is due to the fact that increasing 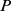 tends to increase the frequency of the pattern–antipattern oscillations of the firing rate and, therefore, to decorrelate the firing rate from the input signal. This is as expected because the memory capacity of the standard Ising–Hopfield model is known to generally decrease due to interference among the stored patterns [42]. For a given value of 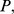 on the other hand, the height of the low-noise peak increases with the frequency 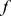 of the signal as this approaches the frequency of the pattern–antipattern oscillations (right graph in Fig. 10). The net result is therefore that SMR is robust for a range of 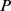 values as far as input signals are of high frequency, while one should expect the low–frequency signals to be poorly processed.

A picture similar to the one in Fig. 1 was reported before in settings that are close to ours here but involving serious restrictive conditions [43]–[45]. In particular, a recent study within the linear and mean-field approximations of the Ising model with — constant and homogeneous — ferromagnetic interactions under an oscillating magnetic field [44], [45] describes resonance behavior when the wiring of connections is not homogeneous. The outcome happens to depend crucially on specific properties of the involved network structure, and the resonance resembles the one in Fig. 1 when the degree distribution obeys a power law 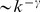 with 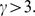 In spite of its interest for other purposes [46]–[48], the relevance of the Ising model on scale-free networks is perhaps questionable within the present context. That is, large values of 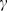 are generally not observed in nature, and the system is physically anomalous due to finite-size effects for 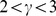 [44]–[48]. On the contrary, it is remarkable in our model that defining its wiring a situation in which all neurons are in principle connected to each other, the intensity of connections is not homogeneous and constantly varies with time. This in fact induces a real complex functionality of the network which is likely to correspond more generally to the one in nature [49]–[53].

**Figure 11:**
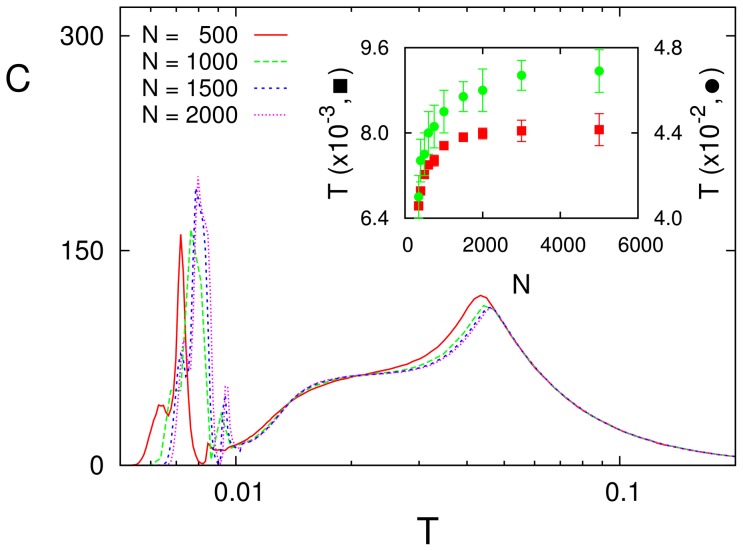
Effect of the network size 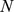
 **on SMR.** The inset shows how the value of 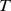 locating the low (circles) and high (squares) noise peaks depends on 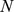. This is for a sinusoidal signal with 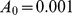 and 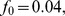 and 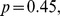 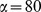 and 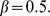

Fig. 11, on the other hand, shows how the results in this paper do not depend essentially on the network size 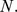 That is, SMR occurs qualitatively the same for a range of sizes, and the value of noise at which the peaks develop depends on 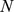 but tends soon to saturate at a constant value. This is interesting because the neural systems that we attempt to describe are far from being *infinite* in the thermodynamic sense but correspond to relatively small values of 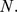


**Figure 12:**
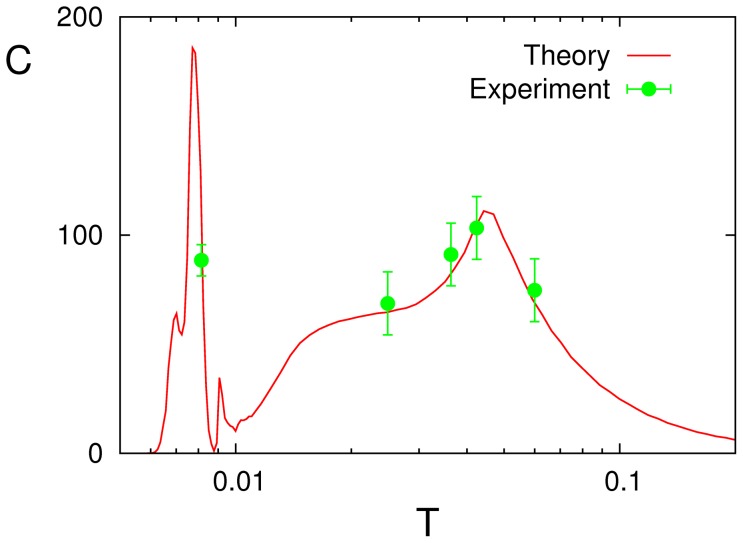
The experimental data (symbols with the corresponding error bars) reported in [**54**] are plotted here against our theoretical prediction (red solid line) corresponding to the case 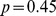
 **in** **Fig. 4** **.** To obtain this fit, the experimental data 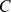 with arbitrary units are multiplied by a factor 180, and the external noise amplitude 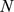 (which is given in dB) needed to be transformed into our intrinsic noise parameter 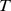 using the nonlinear relationship 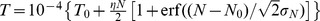 with 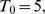 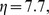 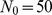
dB, and 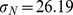
dB.

Finally, we comment on possible experimental realizations of SMR. Some limited data from a psychotechnical experiment [54]–[56] concerning the human cortex were recently interpreted in the light of SMR using a simple model consisting of FitzHugh–Nagumo neurons [57]–[60], which account for adaptive thresholds and fatigue–enduring synapses [21]. This in fact motivated the present study of a similar situation in a complex network. We therefore attempted a new contact between those experimental data and the present model; figure 12 shows the result, which is encouraging. No doubt that further experiments trying to confirm SMR, which will thus clarify the possible existence of intriguing mechanisms as suggested by the model in this paper, will be most welcome.
